# Continuous Labor Support and Person-Centered Maternity Care: A Cross-Sectional Study with Women in Rural Kenya

**DOI:** 10.1007/s10995-021-03259-4

**Published:** 2021-10-19

**Authors:** Gift Kiti, Ndola Prata, Patience A. Afulani

**Affiliations:** 1 School of Public Health, University of California, Berkeley, USA; 2 Bixby Center for Population Health and Sustainability, School of Public Health, University of California, Berkeley, USA; 3 Department of Epidemiology and Biostatics and Institute for Global Health Sciences, University of California, San Francisco, USA

**Keywords:** Person-centered maternity care, Maternity care, Kenya, Sub-Saharan Africa, Continuous support during labor, Birth companionship, Respectful maternity care

## Abstract

**Objective:**

This study assessed whether having continuous support during labor is associated with better person-centered maternity care (PCMC) among women in rural Kenya.

**Methods:**

Data are from a cross-sectional survey with women aged 15–49 years who delivered in the 9 weeks preceding survey completion (N = 865). PCMC was operationalized using a validated 13-item scale, with a summative score developed from responses that capture dignity and respect, communication and autonomy, and supportive care from providers (excluding support from a lay companion). Continuous support was operationalized as the continuous presence of a lay companion (friend or family) during labor. We carried out bivariate analyses using chi-squared and t-tests and ran multivariable linear regression models to examine the association between continuous labor support and PCMC.

**Results:**

The average PCMC score was 24.2 (SD = 8.4) out of a total score of 39. About two-thirds (68%) of women had continuous support during labor. The average PCMC scores among women who had continuous support was 25.7 (SD = 8.4) compared to 21.0 (SD = 7.6) among those who did not have continuous support (p-value ≤ 0.001). After controlling for various confounders this association was still significant (coefficient = 4.0; 95% CI 2.9, 5.2; p-value ≤ 0.001).

**Conclusions:**

Women who have continuous labor support during childbirth are more likely to have improved PCMC. Efforts to promote PCMC should thus include continuous labor support.

## Significance

This paper extends the evidence on the role of continuous labor support on women’s experiences using a validated measure that focuses on aspects of PCMC such as dignity and respect and communication. The findings of this paper can be used to promote continuous labor support as an important aspect of PCMC that promotes other dimensions of PCMC. Improving continuous support will improve women’s overall experiences during childbirth and contribute to improved maternal health.

## Introduction

Globally, approximately 800 pregnancy-related deaths occur daily, with 99% of the deaths coming from low and middle income countries, and 65% from sub-Saharan Africa alone (WHO, 2019). Despite global declines in maternal mortality rates in recent years, it still remains a major challenge in most Sub-Saharan African (SSA) countries. In order to achieve the Sustainable Development Goal target of 70 maternal deaths per 100,000 live-births (WHO, 2019), healthcare services need to be of high quality. A major aspect of quality of care is person-centered maternity care (PCMC). PCMC is “maternity care that is respectful of and responsive to women’s preferences, needs, and values (Afulani et al., 2017, 2018a, b).” PCMC emphasizes the quality of a patient’s experience and encompasses respectful patient-provider communication, provider’s responsiveness, patient engagement and interpersonal treatment (Afulani et al., 2017, 2018a, b).

The World Health Organization (WHO) recommendations for a positive childbirth experience, highlights key domains of PCMC such as—dignity and respect, communication and autonomy and supportive care (Tunçalp et al., 2015). Poor PCMC can result in poor pregnancy outcomes such as improper identification and mismanagement of pregnancy complications (Bohren et al., 2017; Miller et al., 2016). Additionally, poor PCMC leads to low utilization of healthcare services. This is especially because once women share their poor experiences amongst each other, it leads to poor perception of the quality of care (Bohren et al., 2014; Bohren et al., 2017) hence deliveries in unprofessional settings and subsequently high maternal mortality rates. On the contrary, PCMC could contribute effectively to timely healthcare delivery, improved patient-provider communication, increased treatment adherence and consequently lead to better maternal and neonatal outcomes (Fahy, 2012; Miller et al., 2016).

A key aspect of PCMC is emotional and social support during childbirth (Tunçalp et al., 2015). WHO therefore recommends that every woman is offered the option to have a companion of her choice during her labor and childbirth (Tunçalp et al., 2015). Birth companions can offer support through several different ways; these include emotionally by reassurance and praise, through advocacy whereby they ensure the woman’s needs/wishes are well communicated to providers, and informationally by giving updates on the labor progress and advice on coping techniques (Berkman et al., 2000; Bohren et al., 2017; Diamond-Smith et al., 2016). Birth companionship can either be provided by selected companions within the woman’s social network or other non-healthcare professionals such as doulas (Rosen, 2004). There are numerous advantages of having a companion during labor and childbirth, including women experiencing shorter labor spans with more spontaneous vaginal deliveries, lower likelihood of having a caesarean birth or of using pain medication and even lower chances of postpartum depression (Bohren et al., 2017). Additionally, birth companionship is associated with a more positive childbirth experience (Bohren et al., 2017; Campero et al., 1998).

Despite the documented benefits of companionship, previous research has shown that not all women desire a companion during labor citing reasons such as embarrassment for being seen naked by companion, fear of gossip by companions on private matters, or cultural beliefs around the husband/partner not being allowed in the labor or delivery room (Afulani et al., 2018a, b). While most providers acknowledged the importance of continuous labor support, others stated its impossibility due to privacy concerns, inappropriate companion behavior or distrust and lack of confidence in companions (Afulani et al., 2018a, b). In Kenya, some providers were apprehensive about having older women as companions with the fear that they would enforce things done in home deliveries such as giving the laboring woman herbs to hasten labor. These providers were also wary of companions misinforming the laboring women (Afulani et al., 2018a, b). In Brazil, providers did not allow companions because they felt some companions end up being a nuisance to the obstetric team (such as by feeling dizzy or fainting and needing extra attention instead of focussing on the mother) and providers being uncomfortable with the presence of companions as they felt evaluated by their presence (Brüggemann et al., 2014). Other reasons against birth companionship include the small size of labor and delivery wards, absence of private wards, few number of health providers and inadequate health equipment that could lead health providers to carry out improvisations that they would rather non-health providers not see (Brüggemann et al., 2014; Diamond-Smith et al., 2016). Thus, much is yet to be learnt to advocate for companionship.

In 2015, Kenya recorded a maternal mortality ratio of 510 maternal deaths per 100,000 live births compared to a ratio of 12 maternal deaths per 100,000 live births of high-income countries (Alkema et al., 2016). Although Kenya introduced a free maternity services policy, research shows evidence of poor quality care, including poor PCMC, which is contributing to low facility-based childbirth and the high mortality rates. The 2017 confidential enquiry into maternal deaths report in Kenya, which reviewed 484 maternal deaths from major referral hospitals, found that 9 out of 10 women died due to sub-standard care (MOH, Kenya, 2017). Additionally, a study in Kenya showed that 20% of the women reported some form of mistreatment including; non-dignified care, neglect or abandonment, non-confidential care and detention for not paying fees (Abuya et al., 2015). Kenya is one of the countries that has included birth companionship in the country’s guidelines (National Guidelines for Quality Obstetrics and Perinatal Care in Kenya), yet there is very little evidence of its implementation (Afulani et al., 2018a, b). Implementing the recommendations for companionship could potentially improve other aspects of PCMC.

Although prior research suggests that women with continuous labor support are more likely to have better childbirth experiences, until recently, most of these studies measured women’s experiences of care based on satisfaction measures or measures of disrespect and abuse. No study to our knowledge has examined the relationship between continuous labor support and women’s birth experiences using a validated measure which focuses on the positive aspects of PCMC. This is likely because hitherto, there were no such measures. In this paper, we will use the shorter 13-item PCMC scale (Afulani et al., 2019) derived from the longer 30-item PCMC scale (Afulani et al., 2017) to look at the association between having continuous support during labor and women’s experiences of care. The short PCMC scale includes items on dignity and respect, communication and autonomy, and supportive care (Afulani et al., 2019). Notably, there is no item on birth companionship in the short scale (present in the 30-item scale), which makes it appropriate for examining a relationship between continuous support and other aspects of PCMC. We hypothesize that women who had continuous labor support would report higher PCMC scores compared to women without continuous labor support.

## Methods

### Study Setting

The data used in this study was from a larger cross-sectional study examining community perceptions of the quality of maternity care in Migori, described in detail elsewhere (Afulani et al., 2017, 2019). Migori is a rural county in western Kenya, which is divided into 8 sub-counties. It has a population of approximately one million, with an estimated 40,000 annual births (Afulani et al., 2017, 2019) and a fertility rate of 5.3 which is slightly higher than the national average of 3.9 (Health Policy Project, 2015). Migori is one of 15 counties that accounts for over 60% of maternal deaths in Kenya. The estimated maternal mortality ratio stands at 673 maternal deaths per 100,000 live births compared to the national average of 495 (Health Policy Project, 2015). In comparison to the national average of 61%, only 50% of women gave birth in health facilities in Migori county in 2015 (Kenya National Bureau of Statistics et al., 2015). The county has 32, 19 and 4 nurses, clinical officers and doctors respectively per 100,000 people (Health Policy Project, 2015).

### Data Collection

Data were collected from a survey conducted in August and September 2016 with recently delivered women. Eligible women were aged 15–49 years and had delivered in the nine weeks preceding the survey. A multistage sampling approach was used to select women from each of the eight sub-counties, with a target of approximately 200 interviews in each subcounty. This was achieved by first randomly selecting 10 health units from each subcounty and interviewing the first 20 eligible women in each health unit that were available. If the target wasn’t met, then more health units in that sub-county were sampled. The sampling procedures are documented in detail elsewhere (Afulani et al., 2017). Twelve field staff from the study county (one or two from each sub-county), who were trained on the study questionnaire and research ethics, conducted the interviews. The interviews were conducted in English, Swahili, and Luo in private spaces in health facilities or in the homes of the respondents. All participants provided written informed consent after receiving information about the study and were given an incentive of 200 Kenyan shillings (~ $2). Ethical approval for the study was provided by the institutions listed in the ethics statement. About 1000 women (N = 1052) were interviewed, with a response rate above 98%. For this analysis, we excluded 158 women who did not give birth in a health facility, 13 women with missing data on the outcome, 7 women with missing data on the primary exposure and 9 women with missing data on other relevant variables for the analysis to yield an analytic sample of N = 865.

### Measures

#### Dependent Variable (Outcome): Person-Centered Maternity Care (PCMC) Score

The main dependent variable is the PCMC score—a summative score from responses to items in the 13-item PCMC scale (Afulani et al., 2019), which is a shorter version of the previously developed 30-item scale (Afulani et al., 2017). The development and validation of both versions have been previously described (Afulani et al., 2017, 2019). The 13-item PCMC scale was developed based on expert ranking of items in the 30-item scale followed by psychometric analysis using data from rural Kenya (the sample for the current analysis), urban Kenya, India, Ghana. It has high content, construct, and criterion validity as well satisfactory internal consistency reliability (Afulani et al., 2019). The Cronbach’s alpha for the dataset in this study is 0.8. Each item is on a 4-point frequency response scale—0: “no, never,” 1: “yes, a few times,” 2: “yes, most of the time,” and 3: “yes, all the time.” The responses for the 13 questions are combined to create a summative score which ranges 0 to 39, with lower scores implying poorer PCMC.

### Independent Variable

#### Exposure

The independent variable is continuous support during labor. The survey question for this variable asked; “Were you allowed to have someone you wanted (from outside of staff at the facility, such as family or friends) to stay with you during labor?” To create a measure of continuous labor support, we recoded “no, never,” and “yes, a few times,” into No (0) and the “yes, most of the time,” and “yes, all the time” into Yes (1). The inclusion of the “yes, a few times,” into the no category was is to emphasize on “continuous” support. The 5 respondents who responded “I did not want someone to stay with me” were also conservatively recoded into “Yes,” which assumes that someone who said that “they did not want a support person” would have been allowed one if they so desired (Afulani et al., 2017).

### Covariates

Covariates included socio-demographic (age, marital status, parity, education, employment, household wealth, and subcounty) and health system i.e. facility and provider related factors (type of delivery facility, type and sex of delivery providers) that might be associated with the dependent variable based on existing literature (Afulani et al., 2017, 2018a, b). We also controlled for postpartum length and place of interview as they have been shown to be associated with women’s reports of their experiences (Afulani et al., 2017).

## Analysis

The initial analyses involved descriptive statistics for the sample-means for continuous variables and proportions for categorical variables. Next we examined the bivariate associations between the independent variable and the dependent variable through cross tabulations of the mean PCMC scores by the continuous labor support variable. We ran linear regressions as the PCMC score is a normally distributed continuous variable (Martinez, 2007), and calculated robust standard errors to account for clustering (MacKinnon & White, 1985) at the health unit. We included subcounty as a fixed effect. We built the multivariable models starting with a bivariate model and sequentially added other covariates that were significant in the bivariate models. Model fit test (AIC values) were used to select variables in the final multivariate models. All analyses were conducted in R studio version 3.6.1.

## Results

Table 1 shows the univariate distributions of the study demographic variables. The average age is about 25 years, with 19% being less than 20 years old. Approximately 78% are married, with average parity of three; 27% have more than four children. About 56% have only primary education or less and 25% are employed. The distribution of mothers between the subcounties is relatively similar with majority coming from the Suna West subcounty. About half (46%) gave birth in public hospitals, 41% in health centers and about 13% delivered in private facilities. Close to 60% of the interviews occurred outside a health facility.

**Table 1:** Demographic characteristics of study participants

	No	(%)
*Total* N	865	100
*Marital status*		
Married	678	78.38
Not married	187	21.62
*Maternal age (years)*		
15–19	162	18.73
20–29	501	57.92
30–48	202	23.35
Mean age (SD)	865	24.9 (5.9)
*Maternal education*		
No school/not finished primary	176	20.35
Primary	310	35.84
Post-primary/vocational/secondary	270	31.21
College or above	109	12.60
*Wealth quintile*		
Poorest	373	43.12
Middle	134	15.49
Richest	358	41.39
*Do work for pay*		
No	649	75.03
Yes	216	24.97
*Number of births*		
1.0	289	33.41
2.0	181	20.92
3.0	160	18.50
4.0 or more	235	27.17
*Mean parity* (SD)	865	2.8 (2.0)
*Place of interview*		
Health facility	351	40.58
In the community/a home	514	59.42
*Time of interview*		
Less than 5 weeks	426	48.74
5 weeks or more	448	51.26
*Post-partum length*		
Less than 5 weeks	422	48.79
5 weeks or more	443	51.21
*Subcounty*		
Suna East	156	18.03
Suna West	85	9.83
Rongo	122	14.10
Awendo	103	11.91
Kuria West	110	12.72
Kuria East	115	13.29
Nyatike	100	11.67
Uriri	74	8.55
*Delivery facility type*		
Public hospital	396	45.78
Health center	359	41.50
Mission/private facility	110	12.72
*Delivery provider sex*		
Male	325	37.57
Female	506	58.50
Both	34	3.93
*Delivery provider*		
Nurse/midwife	646	74.68
Doctor/clinical officer	136	15.72
Non-skilled attendant	21	2.43
1plus skilled providers	62	7.17
*Company type to health facility*^a^		
Partner-husband	250	28.90
Mother-in-law	241	27.86
Mother	101	11.68
Sister/sister-in-law	164	18.96
Friend/neighbor	113	13.06

^**a**^Companion options were select all that apply hence not mutually exclusive and don’t add to 100%

Characteristics associated with continuous labor support and PCMC are shown in Table 2. Approximately 68% of the women had continuous support. Women with higher education, higher wealth, and those who work for pay were more likely to have continuous support than women with lower education, lower wealth, and without salaried jobs respectively. Continuous support was also more common among women with lower parity, those who delivered in a health center, and those assisted by a female provider or a non-skilled attendant compared respectively to their reference groups. The average PCMC score is 24.2 (SD = 8.4) out of a total score of 39 and 25.7 among women with continuous support compared to 21.0 among those with no continuous support. Women who are married, educated, wealthier and hold salaried jobs on average had higher PCMC than women who are unmarried, less educated, poorer and without salaried jobs respectively. The mean PCMC score for unemployed women is 23 compared to 27 for those employed. On average, women from Kuria West subcounty had the highest PCMC scores (31.5).

**Table 2:** Bivariate Distribution of study variables by exposure and outcome

	Proportion with continuous support n = 591 (68%)	Pearson chi-squared P-value	PCMC score Mean ± SD n = 865	F-test/t-test P Value
*Continuous support*				
No	–		21.0 ± 7.6	< 0.001
Yes	–		25.7 ± 8.4	
*Marital status*				
Married	457(67.4%)		24.6 ± 8.3	0.0022
Not married	134 (71.8%)	0.31	22.5 ± 8.7	
*Maternal age* (years)				
15–19	118 (72.8%)	0.14	23.0 ± 8.8	0.1334
20–29	345 (68.9%)		24.6 ± 8.3	
30–48	128 (63.4%)		24.2 ± 8.3	
*Maternal education*				
No school/not finished primary	97 (55.1%)	< 0.001	24.1 ± 9.3	0.11
Primary	219 (70.6%)		23.4 ± 8.3	
Post-primary/vocational/secondary	189 (70.0%)		24.5 ± 8.2	
College or above	86 (78.9%)		25.6 ± 7.8	
*Wealth quintile*				
Poorest	231 (61.9%)	< 0.001	23.1 ± 8.5	0.0019
Middle	92 (68.7%)		24.0 ± 8.4	
Richest	268 (74.9%)		25.3 ± 8.3	
*Do work for pay*				
No	421 (64.9%)	< 0.001	23.3 ± 8.3	< 0.001
Yes	170 (78.7%)		26.9 ± 8.3	
*Number of births*				
1.0	215 (74.4%)	0.0022	23.7 ± 8.8	0.0588
2.0	132 (72.9%)		25.4 ± 8.3	
3.0	100 (62.5%)		24.8 ± 7.7	
4.0 or more	144 (61.3%)		23.4 ± 8.4	
Place of interview				
Health facility	231 (65.8%)	0.22	25.4 ± 8.5	< 0.001
In the community/a home	360 (70.0%)		23.3 ± 8.3	
*Post-partum length*				
Less than 5 weeks	292 (69.2%)	0.64	28.1 ± 9.3	0.0313
5 weeks or more	299 (67.5%)		23.8 ± 8.3	
*Subcounty*				
Suna East	113 (72.4%)	0.00	26.6 ± 8.2	< 0.001
Suna West	34 (40.0%)		24.5 ± 7.0	
Rongo	102 (83.6%)		21.0 ± 8.2	
Awendo	71 (68.9%)		22.0 ± 5.3	
Kuria West	92 (83.6%)		31.5 ± 7.5	
Kuria East	39 (33.9%)		20.0 ± 8.5	
Nyatike	85 (85.0%)		24.9 ± 8.9	
Uriri	55 (74.3%)		21.6 ± 6.3	
*Delivery facility type*				
Public hospital	255 (63.7%)	0.0012	23.3 ± 8.5	< 0.001
Health center	271 (75.3%)		24.4 ± 8.3	
Mission/private facility	70 (63.1%)		26.8 ± 8.3	
*Delivery provider sex*				
Male	202 (62.2%)	0.0101	24.5 ± 8.4	0.0065
Female	365 (72.1%)		23.7 ± 8.5	
Both	24 (70.6%)		28.3 ± 7.2	
*Delivery provider*				
Nurse/midwife	454 (70.3%)	0.0093	23.9 ± 8.5	0.20
Doctor/clinical officer	88 (64.7%)		24.4 ± 8.5	
Non-skilled attendant	17 (81.0%)		25.9 ± 8.3	
1plus skilled providers	32 (51.6%)		26.0 ± 7.6	

Percentages under proportion with continuous support column are from row totals, so proportion without support for each row can be calculated by subtracting from 100

The regression results for continuous labor support and PCMC is shown in Table 3. In crude analysis (model 1) we see that women who had continuous labor support scored 4.67 points higher on the PCMC scale than those without. When we adjust for companion type (model 2) the coefficient estimate goes slightly higher to 4.78. Controlling for demographic factors causes the estimate drop slightly to 3.85 (model 3). The full model (model 4) adjusts for health system factors and we see that net of other factors, women who had a continuous labor support scored 4.04 points higher on the PCMC scale than those lacking continuous labor support. All four models showed statistically significant associations (p < 0.001).

**Table 3:** Multivariate OLS regression of continuous labor support and PCMC, N = 865

					Model 1: Unadjusted		Model 2: Adjusted for companion type		Model 3: Demo-graphics added		Model 4: Health system factors added	
					β (95%CI)		β (95%CI)		β (95%CI)		β (95%CI)	
*Continuous labor support*												
No					Ref		Ref		Ref		Ref	
Yes					4.67*** (3.50, 5.84)		4.78*** (3.61, 5.95)		3.85*** (2.69, 5.01)		4.04*** (2.87, 5.20)	
*Companion type*												
*Partner-husband*												
No							Ref		Ref		Ref	
Yes							1.36* (0.13, 2.60)		0.98 (− 0.18, 2.14)		0.98 (− 0.17, 2.14)	
*Mother-in-law*												
No							Ref		Ref		Ref	
Yes							2.32*** (1.02, 3.61)		1.40 0.14, 2.66)		1.54* (0.28, 2.80)	
*Mother*												
No							Ref		Ref		Ref	
Yes							− 0.96 (− 2.73, 0.80)		− 0.18 (− 1.98, 1.61)		− 0.14 (− 1.93, 1.64)	
*Sister/sister-in-law*												
No							Ref		Ref		Ref	
Yes							0.49 (− 0.95, 1.93)		0.84 (− 0.48, 2.16)		0.93 (− 0.39, 2.25)	
*Friend/neighbor*												
No							Ref		Ref		Ref	
Yes							1.91* (0.28, 3.55)		2.48* (0.95, 4.01)		2.63*** (1.10, 4.15)	
*Age*												
									0.03 (− 0.09, 0.14)		0.03 (− 0.82, 0.15)	
Parity												
									− 0.20 (− 0.54, 0.13)		− 0.23 (− 0.56, 0.11)	
*Marital status*												
Married									Ref		Ref	
Not married									− 0.75* (− 2.17, 0.67)		− 0.82 (− 2.23, 0.60)	
*Wealth Quintile*												
*Poorest*									Ref		Ref	
Middle									0.56 (− 0.91, 2.03)		0.63 (− 0.84, 2.09)	
Richest									2.2*** (0.99, 3.42)		2.08** (0.83, 3.32)	
*Do work for pay*												
No									Ref		Ref	
Yes									1.29*** (0.04, 2.55)		1.14** (− 0.12, 2.40)	
*Subcounty*												
Awendo									Ref		Ref	
Suna East									4.05*** (2.22, 5.88)		0.25*** (1.51, 5.24)	
Suna West									3.18*** 1.71, 6.06)		8.55*** (1.66, 6.05)	
Rongo									− 2.68** (− 4.67, − 0.68)		1.64* (− 4.17, 0.11)	
Kuria West									8.68*** (6.62,10.7)		− 2.14*** (6.45, 10.7)	
Kuria East									− 0.13 (− 2.19, 1.94)		3.38 (− 1.87, 2.37)	
Nyatike									1.75 (− 0.31, 3.81)		3.85 (− 0.43, 3.71)	
Uriri									− 0.64 (− 2.84, 1.56)		− 0.62 (− 2.82, 1.59)	
*Place of interview*												
Health facility											Ref	
In the community/a home											− 1.29* (− 2.33,− 0.25)	
*Post-partum length*												
Less than 5 weeks											Ref	
5 weeks or more											− 0.53 (− 1.53,0.46)	
*Delivery facility type*												
*Public hospital*											Ref	
Health center											0.56 (− 0.62, 1.74)	
Mission/private facility											2.13** (0.54, 3.72)	
*Delivery provider sex*												
Male											Ref	
Female											0.09 (− 1.13, 1.32)	
Both											1.72 (− 0.1.29, 4.73)	
*Delivery provider*												
Nurse/midwife											Ref	
Doctor/clinical officer											0.79 (− 0.84, 2.41)	
Non-skilled attendant											2.28 (− 0.95, 5.51)	
1plus skilled providers											1.45 (− 0.91, 3.80)	

*CI* 95% Confidence intervals

^*^p < 0.05; **p < 0.01; ***p < 0.001

In the model accounting for type of companion only (model 2), women supported by a partner-husband, mother-in-law, or friend had slightly higher PCMC scores than those not supported by each of these companion types. However, only the presence of a friend or mother-in-law were statistically significant in the final model with 2.63 and 1.54 higher points respectively. In the final model, women in the highest wealth quintile had about 1.5 points higher PCMC scores than those in the lowest quintiles, and employed women had about 1.1 points higher PCMC scores than those who are not employed. Women in Kuria West still had the highest PCMC scores which were 8.7 points higher than the reference (women in Awendo sub-county). In addition, women who delivered in mission/private facilities scored about 2 points higher on the PCMC scale than those who delivered in public hospitals.

## Discussion

We examined the association between having continuous support during labor and other aspects of PCMC using survey data from women who recently delivered in a rural county in Kenya. The analysis, supports our hypothesis that women who had continuous labor support would have higher PCMC scores than women without continuous labor support. Our findings thus add to the evidence that continuous support during labor by a companion other than the health providers increases the likelihood of receiving person centered maternity care.

Our findings are consistent with prior studies. For example, a study in three public facilities in Lebanon, Egypt and Syria found that the presence of labor companions made communication between health care providers, women and their families much better. Women in the study also reported feeling “dignified” and “strong” in the presence of companions (Kabakian-Khasholian et al., 2019). Other studies have found continuous support during labor to increase a woman’s satisfaction with maternal healthcare services (Bohren et al., 2017; Srivastava et al., 2015). Similarly, a study in Lucknow, India looking at the association between women’s support (instrumental, informational, or emotional) and their mistreatment experiences during childbirth showed that having a companion decreased mistreatment (Diamond-Smith et al., 2016).

Our finding that women accompanied by their mothers-in-law were more likely have higher PCMC scores is corroborated by several studies in South Asia that have highlighted the mothers-in-law supportive role in some maternal health care practices (Barua & Kurz, 2001; Diamond-Smith et al., 2012; Simkhada et al., 2010). Besides, in many cultures most mothers-in-law play the advocative role during childbirth not only for the sake of their daughters-in-law but especially for the new offspring of the family—the infant. Additionally, married women were more likely to receive continuous labor support compared to unmarried women. Given that most married women are likely to be living with their husband’s families in the cultural context of Migori it does not come as a surprise that the mother-in-law would accompany their daughter-in-law during labor. Not being accompanied by a mother-in-law in this context may thus indicate an unsupportive relationship with the mother in-law. A prior study in India showed that women who received informational support from mothers/mothers-in-law were significantly more likely to report lower mistreatment scores compared to women not receiving this support (Abuya et al., 2015; Diamond-Smith et al., 2016). While our findings show that the presence of a friend/neighbor positively influenced PCMC, Diamond-Smith’s India study found that receiving emotional support from a friend/neighbor/other family member was negatively associated with PCMC (indicated by a higher mistreatment score). This difference may be due to the different cultural contexts whereby in India, age-hierarchies are much stronger, and given friend/neighbors are more likely to be younger, they may exert little influence in this context. Future studies exploring how and why the characteristics of support persons influence PCMC in different contexts are needed.

The findings of this study on the predictors of PCMC based on the shorter 13-item PCMC scale are consistent with the longer version 30-item version. In both studies, employed women and those from wealthier households reported higher PCMC scores than those who are not employed and from poor households. Additionally, women who delivered in health centers and private facilities reported higher PCMC scores compared to those that delivered in public hospitals (Afulani et al., 2017). Our finding that that being married was associated with a higher PCMC score is consistent with previous qualitative studies that have shown that compared to married women, adolescent or unmarried women tend to experience more mistreatment especially in cases when the pregnancy was acquired outside wedlock (Afulani et al., 2018a, b; Bohren et al., 2015). The association between these sociodemographic factors and PCMC introduces some endogeneity in the relationship between the continuous support and PCMC, given these factors are also associated with continuous support. We attempted to address this by controlling for sociodemographic factors in our analysis. The multivariate analysis suggests that continuous support influences PCMC independent of these sociodemographic characteristics. Future experimental studies where women are randomly assigned to the exposure are however needed to fully isolate the effect of continuous support on PCMC.

## Limitations and Strengths

Study limitations should be noted. First, both continuous support and PCMC scale are based on self-reporting which could potentially result in social desirability bias. This is especially true for women who were interviewed in the health facility and close to the time of birth, as previous studies suggest that women are less likely to report negative experiences when interviewed in the health facility and immediately following childbirth due to the joy of having a baby (Kruk et al., 2014). The data were also collected up to 9 weeks after the birth which could have led to recall bias. Secondly, since this is a cross-sectional study, the outcome and exposure were collected at the same time. The findings of this study may also not be generalizable to the whole country or other settings since the data are from one rural county in Kenya. Another potential limitation is the use of the shorter 13-item PCMC scale which could have missed other relevant characteristics of PCMC that the women could have experienced such as lack of confidentiality and verbal or physical abuse. Finally, unlike qualitative data, quantitative data fails to offer the nuances useful in understanding the various pathways by which birth companionship affects quality of care to help shape interventions.

Despite these limitations, our research is the first quantitative study to examine the relationship between continuous labor support and women’s birth experiences using a validated measure. Secondly our research focusses on the positive aspects of PCMC i.e. dignity and respect, communication and autonomy, and supportive care unlike most previous studies that have examined it from the perspective of abuse and disrespect or mistreatment. Thirdly, our study extends the literature on birth companionship and women’s experiences of care. Lastly, our study makes valuable contributions to the existing literature on the disparities in PCMC especially in low resource settings. The findings are also applicable in high resource countries where disparities in PCMC contributes to disparities in adverse maternal outcomes.

## Conclusion

This study has shown that despite the inequities in healthcare delivery in Kenya, the presence of a birth companion during labor could result in a better overall PCMC. This work adds important insights that can be used to improve practice and policies on quality of care in both low-resource and high-resource settings. Given that many health systems in low resource settings are unable to provide continuous support during labor because of privacy concerns, structural interventions are necessary to ensure labor wards are conveniently arranged to accommodate birth companions. These interventions could be in the form of redesigned or expanded and partitioned wards, as well as the construction of private wards, so as to accommodate preferred companions by the mother while also ensuring privacy. Appropriate interventions to sensitize women, family and providers about the role of continuous support during labor are also needed. Furthermore, our study has shown that the quality of care in public facilities is still lacking. Targeted PCMC interventions in public health facilities are therefore necessary to ensure equity in the quality of care delivered. The findings underscore the need for interventions to improve continuous labor support as well as other aspects of person-centered maternity care in health facilities in low resource settings. This would help increase utilization of maternal health services and lower maternal and neonatal morbidity and mortality.

## Data Availability

The data analyzed for the manuscript are available from the corresponding author on reasonable request.

## Consent for Publication

Not applicable.
